# Supplementary material for: GlyContact analyzes glycan 3D structures at scale
Source: Nat Commun. 2025 Dec 12;16:11136. doi: 10.1038/s41467-025-67590-y (PMC12706034; doi:10.1038/s41467-025-67590-y)
Supplement: Supplementary file 1 — Supplementary Information [file 41467_2025_67590_MOESM1_ESM.pdf]

GlyContact analyzes glycan 3D structures at scale

Luc Thomès[1], Roman Joeres[2,3,4], Zeynep Akdeniz[2,3], Daniel Bojar[2,3,*]

[1]University Lille, CHU Lille, ULR 7364 - RADEME - Maladies RAres du DÉveloppement embryonnaire et du Métabolisme, 59000 Lille, France.

[2]Department of Chemistry and Molecular Biology; University of Gothenburg; Gothenburg, 405 30; Sweden

[3]Wallenberg Centre for Molecular and Translational Medicine; University of Gothenburg; Gothenburg, 405 30; Sweden

[4]Saarbruecken Informatics Campus, Saarland University, Saarbruecken, 66123; Germany

[*]Corresponding author

Supplementary Figures

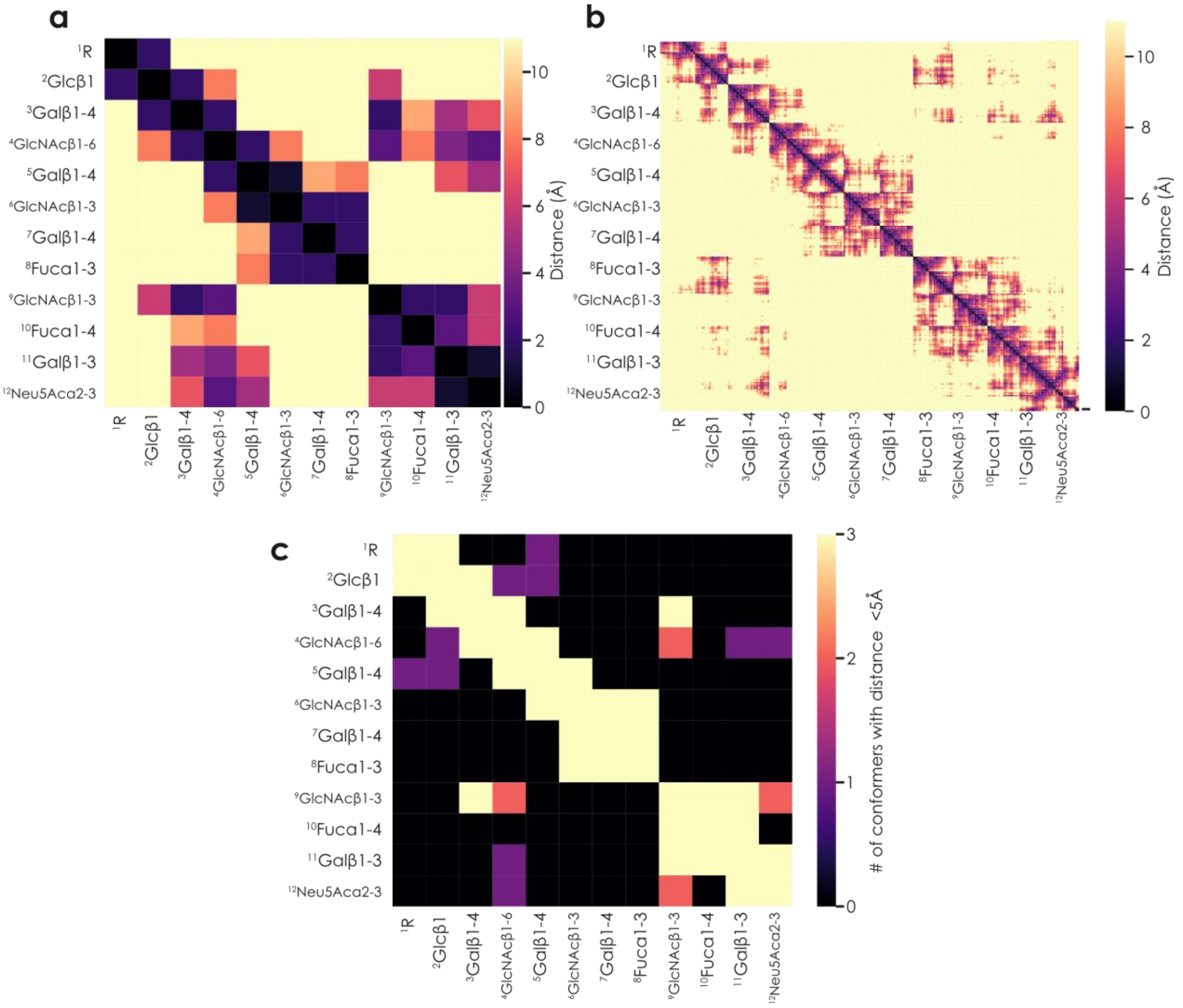

Supplementary Figure 1. Contact maps for the glycan Fucα1-3(Galβ1-4)GlcNAcβ1-3Galβ1-4GlcNAcβ1-6(Neu5Acα2-3Galβ1-3(Fucα1-4)GlcNAcβ1-3)Galβ1-4Glc. a-c) For the conformer beta_2 (a-b) or all conformers (c), we calculated their monosaccharide- (a, c) or atom-level (b) contact maps, using the *glycontact.process.make_monosaccharide_contact_table* and *glycontact.process.make_atom_contact_table* functions from GlyContact, respectively. For (c), we then used the *glycontact.process.inter_structure_frequency_table* function to analyze in how many conformers two monosaccharides were spatially adjacent.

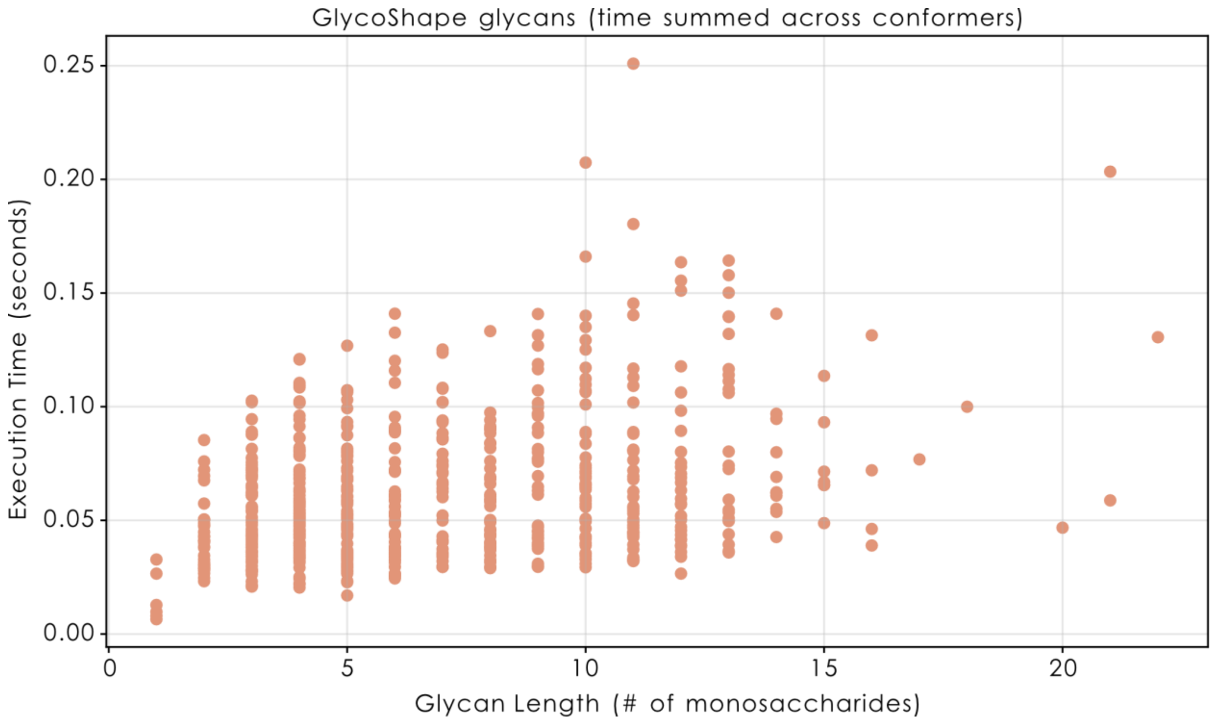

Supplementary Figure 2. Efficient processing of glycan PDBs with GlyContact. For all 717 glycans with deposited PDBs on GlycoShape, we processed all their conformers via the *glycontact.process.annotation_pipeline* function, timed the execution, and show the execution time (summed across conformers) for all glycans. Glycan length is calculated by number of monosaccharides.

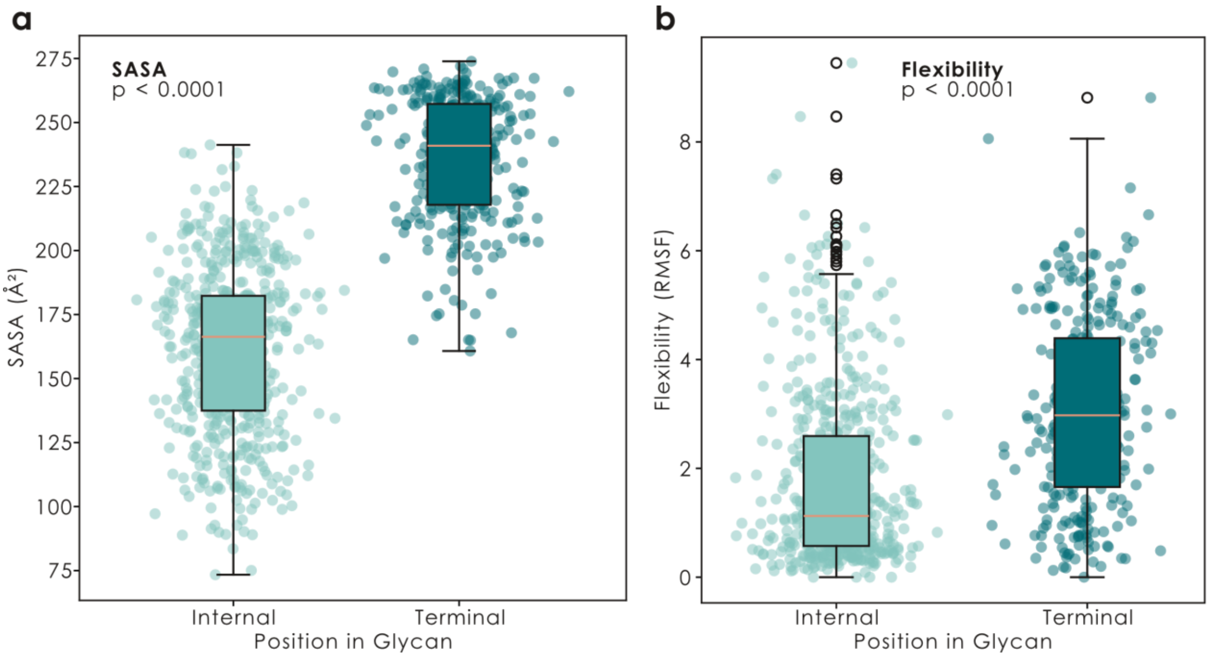

Supplementary Figure 3. Terminal monosaccharides are more accessible and more flexible. a-b) For the monosaccharide galactose, we compared the SASA (a) or flexibility (b) values of all occurrences of galactose, across all GlycoShape structures (averaged across conformers, weighted by their proportions), and compare galactose in terminal positions (i.e., non-reducing ends) with internal positions. The data are shown as box plots (line indicating the median, box edges indicating the 25th and 75th percentile, whiskers indicating the 95% confidence interval, and black circles indicating outliers), as well as an overlaid scatter plot of the actual values, with added horizontal jitter for visibility. Statistical significance was established with a two-tailed Mann-Whitney U test (a: $p = 4.3e-122$; b: $p = 1.2e-26$).

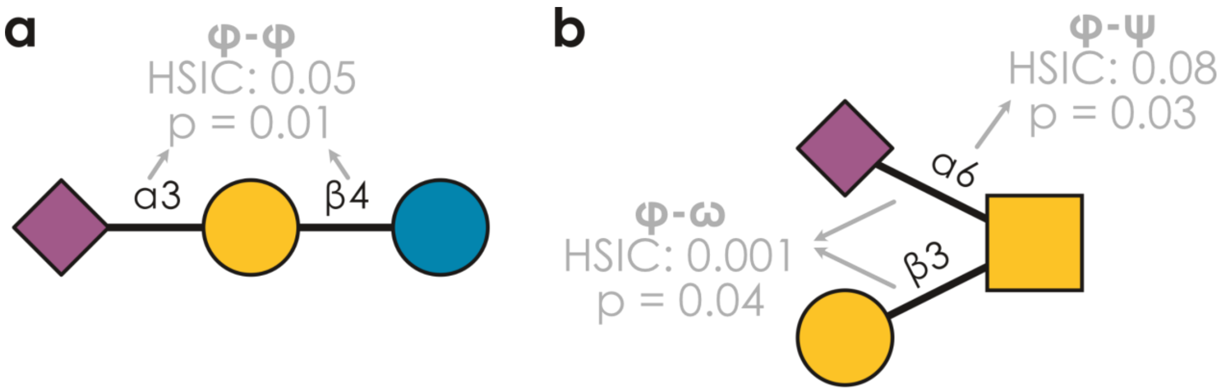

Supplementary Figure 4. Torsion-torsion dependencies can be extracted via non-linear correlation analysis. a-b) For the example structures Neu5Acα2-3Galβ1-4Glc (a) and Galβ1-3(Neu5Acα2-6)GalNAc (b), we used the *glycontact.process.analyze_torsion_torsion_correlations* function to probe the non-linear correlation of all pairs of glycosidic torsion angles for that sequence. Briefly, this used the Hilbert-Schmidt Independence Criterion (HSIC) on the respective torsion angle values across conformers, which is only 0 for truly independent variables, and a subsequent two-sided χ^2 test to statistically test the obtained HSIC against 0. Significant correlations are shown with their HSIC values and p-values.

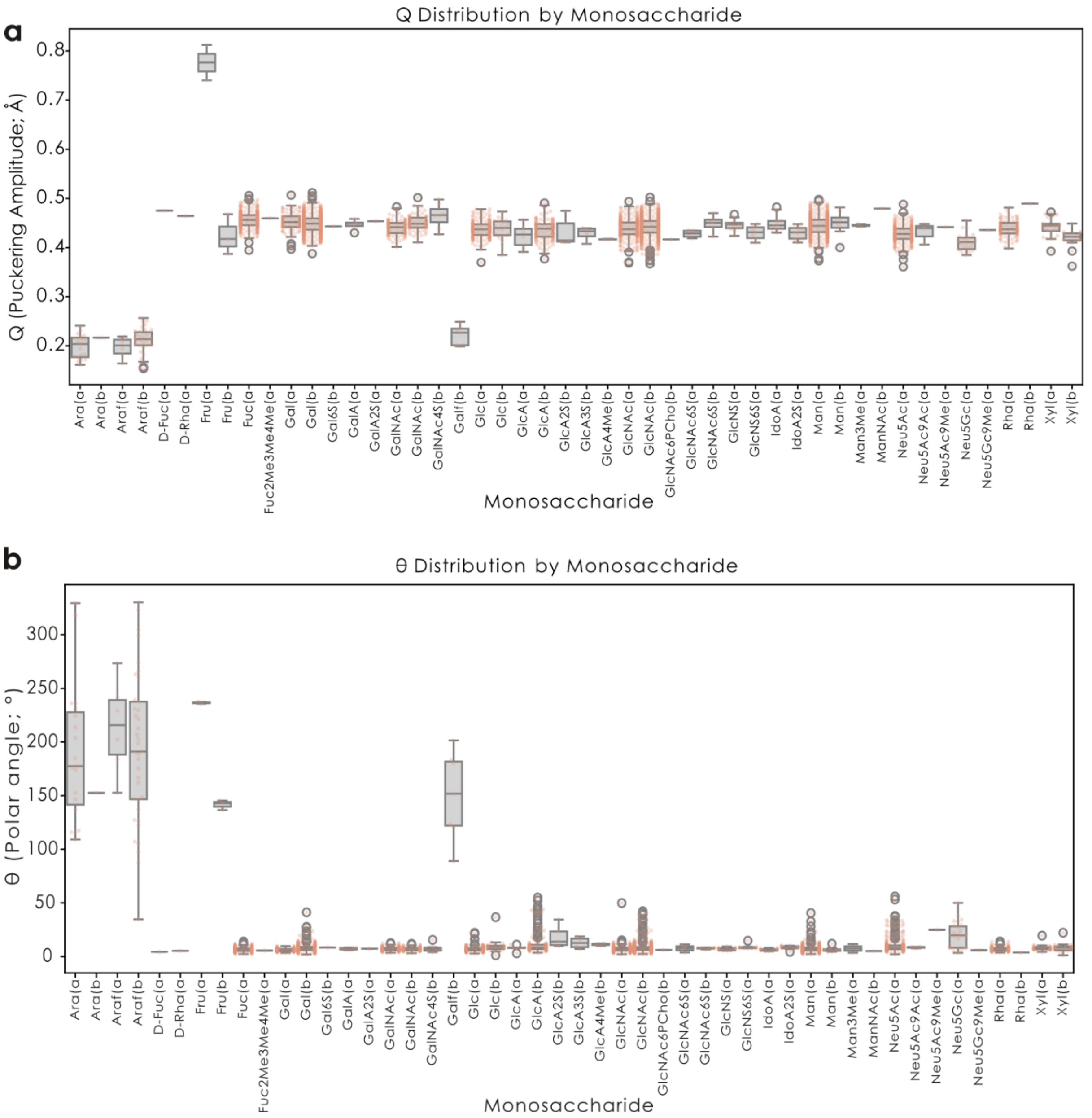

Supplementary Figure 5. Ring puckering shows similar ranges across monosaccharides. a-b) For all glycan structures on GlycoShape, we calculated all puckering amplitudes (Q, a) and polar angles (θ, b) and plotted them here per monosaccharide. The data are shown as box plots (line indicating the median, box edges indicating the 25th and 75th percentile, whiskers indicating the 95% confidence interval, and gray circles indicating outliers), as well as an overlaid scatter plot of the actual values, with added horizontal jitter for visibility.

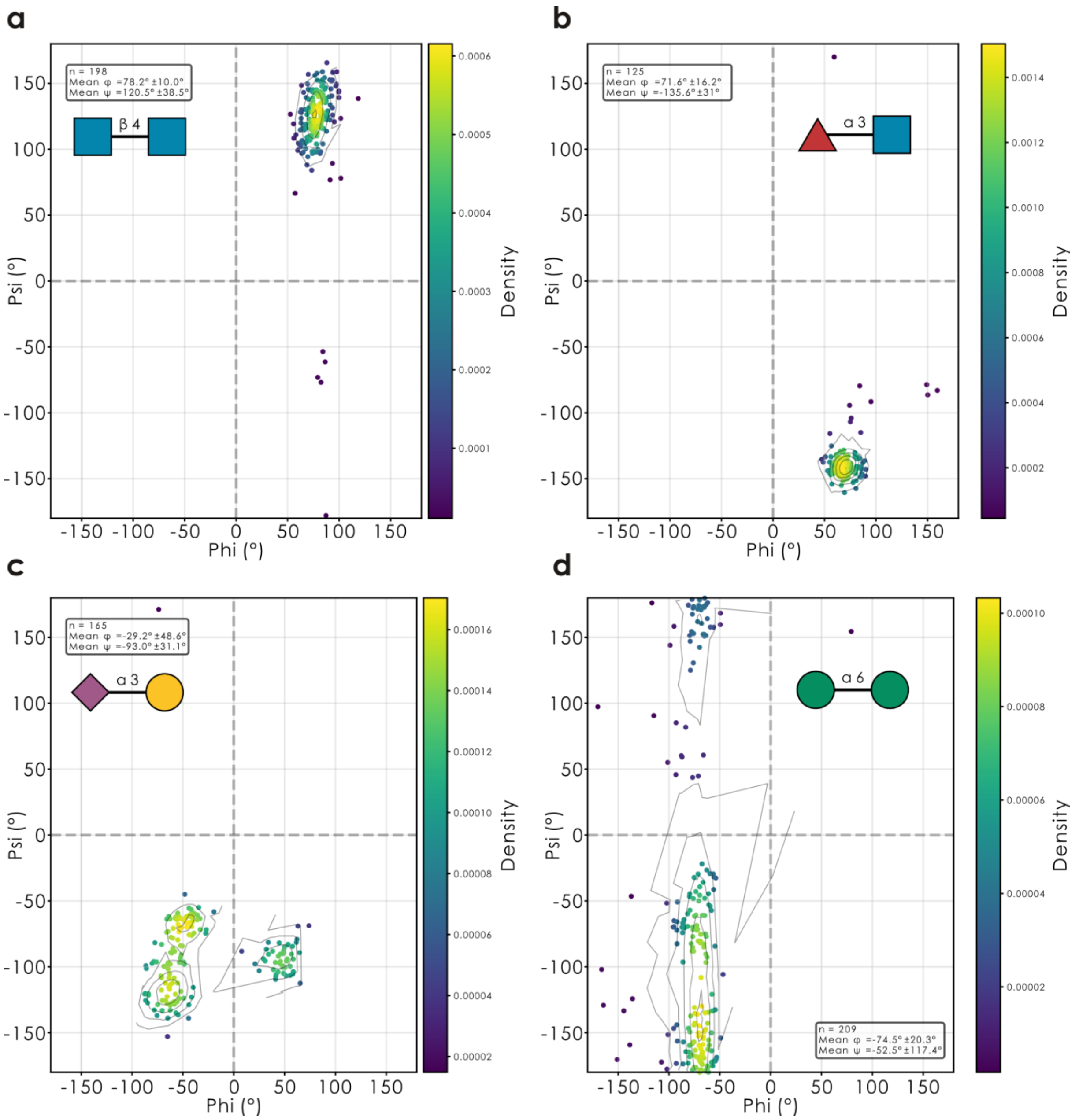

Supplementary Figure 6. GlyContact-calculated torsion angles conform to common Ramachandran distributions. a-d) For the example disaccharides GlcNAcβ1-4GlcNAc (a), Fucα1-3GlcNAc (b), Neu5Acα2-3Gal (c), and Manα1-6Man (d), we extracted all their occurrences from all glycan structures available on GlycoShape and calculated their torsion angles (φ, ψ) via the *glycontact.process.get_glycosidic_torsions* function within GlyContact, which is shown here via Ramachandran plots with the contours derived from a Gaussian kernel density estimate. Both number of occurrences and angle mean values are depicted in the respective panel. The entire workflow is available via the *glycontact.visualize.ramachandran_plot* function.

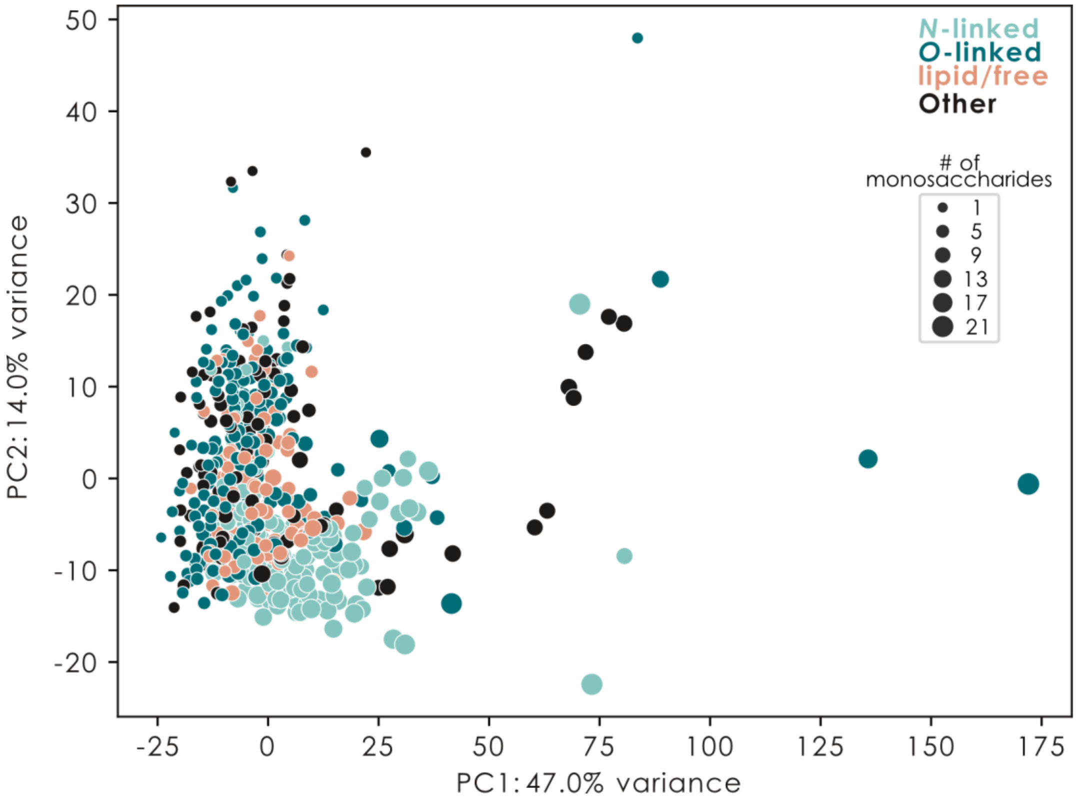

Supplementary Figure 7. Size distribution of glycans explains their clustering by alignment profile. Using the same alignment distance matrix as in Fig. 2a, we here depict a principal component analysis (PCA) for all glycans with structural information, in which color indicates glycan class and the size of a point its length in monosaccharides. Shown are the first two principal components, with their percent variance explained.

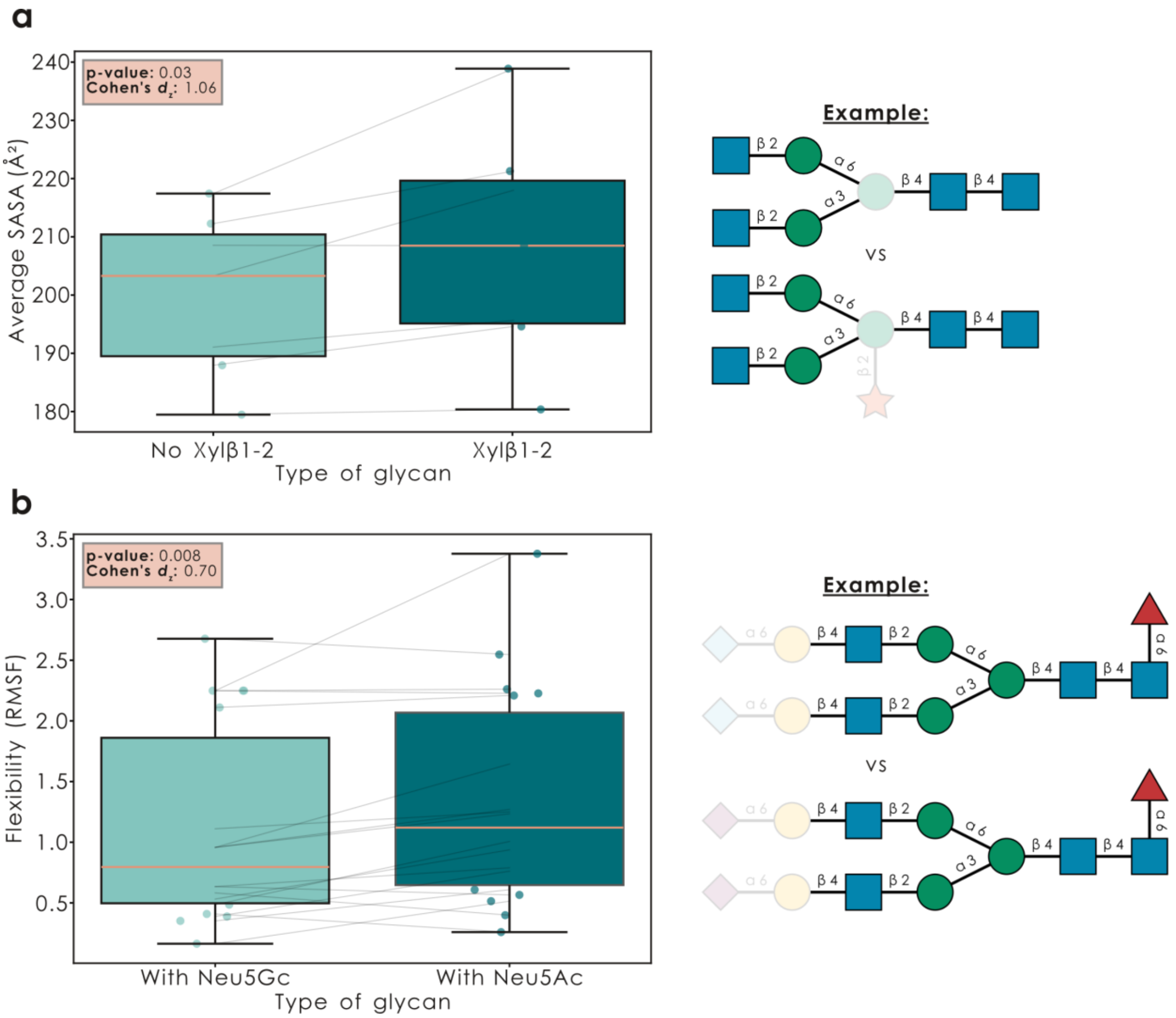

Supplementary Figure 8. Xylose addition opens up plant *N*-glycans and Neu5Ac increases glycan flexibility. a-b) For the example of Xylβ1-2 occurrence in *N*-glycans, typical in plants, (a) and Neu5Ac vs Neu5Gc in all GlycoShape glycans (b), we gathered all 'twins' (pairs of sequences that only differed in the presence/absence of Xylβ1-2 or in Neu5Ac vs Neu5Gc, respectively) for which we had structural data from GlycoShape (a, n = 7; b, n = 19) and compared their average SASA (a) or flexibility (b) values (excluding the considered motif and its attachment site). Results are shown as box plots (line indicating the median, box edges indicating the 25th and 75th percentile, and whiskers indicating the 95% confidence interval), as well as an overlaid scatter plot of the actual values, with added horizontal jitter for visibility. Statistical testing involved a paired two-tailed t-test and Cohen's d_z as an effect size for paired samples. The entire workflow is available via the *glycontact.visualize.find_difference* function.

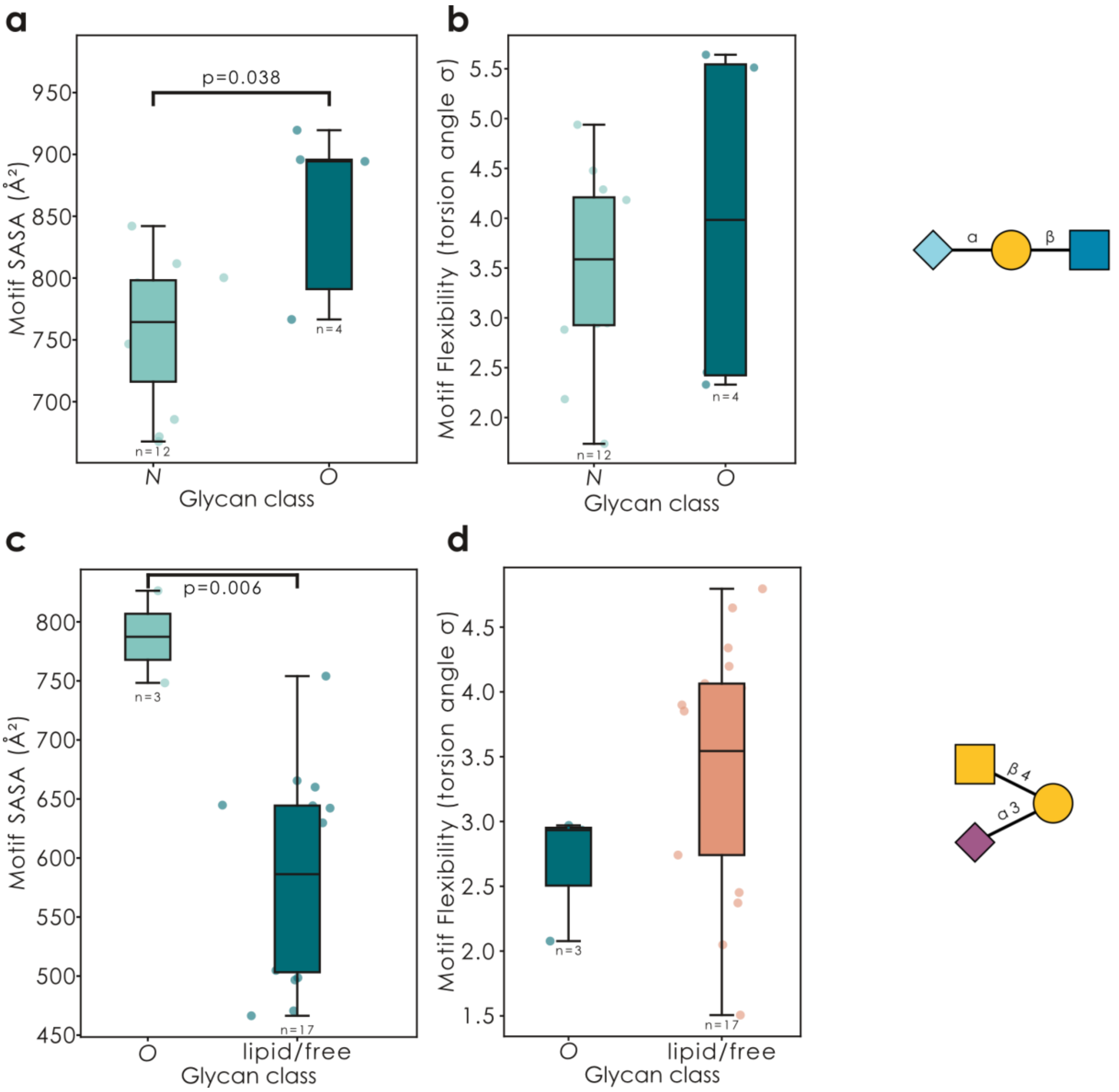

Supplementary Figure 9. Glycan motifs exposed by different glycan classes exhibit different structural characteristics. a-d) For Neu5Gc-LacNAc (Neu5Gcα2-?Galβ1-?GlcNAc; a-b) and Sd[a] (c-d) in GlycoShape glycans, we summed monosaccharide-level SASA values for each motif (a, c) and averaged their torsion-based flexibility (b, d). Then, we grouped glycans by glycan class and analyzed differences across classes by an ANOVA, followed by Tukey's HSD post-hoc test. The number of analyzed motif instances is provided under each box plot.

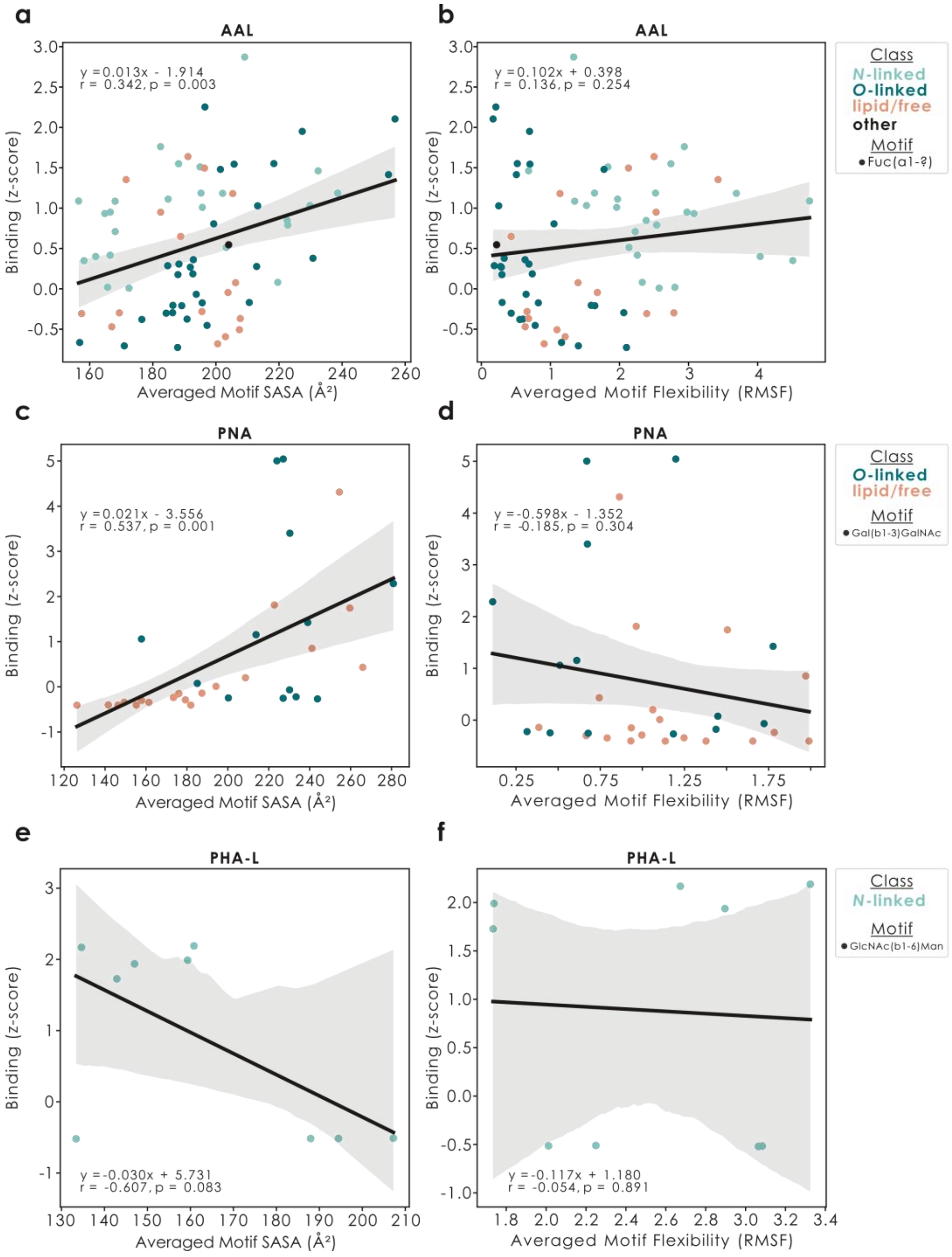

Supplementary Figure 10. Structural motif properties affect lectin binding. a-f) For the lectins AAL (a-b), PNA (c-d), and PHA-L (e-f), we used the z-score transformed binding data from glycowork (v1.6) and correlated it with either the averaged SASA (a, c, e) or flexibility (b, d, f) of the literature-known binding motif in each glycan that (i) carried the binding motif, (ii) had binding data, and (iii) was deposited on GlycoShape. On top of the data points as a scatter plot (colored by glycan class), we then drew a linear least-squares regression line (line representing the mean and including 95% confidence band) as well as the regression equation, the Pearson's correlation coefficient r, and the p-value of a two-sided t-test of the regression coefficient against zero.

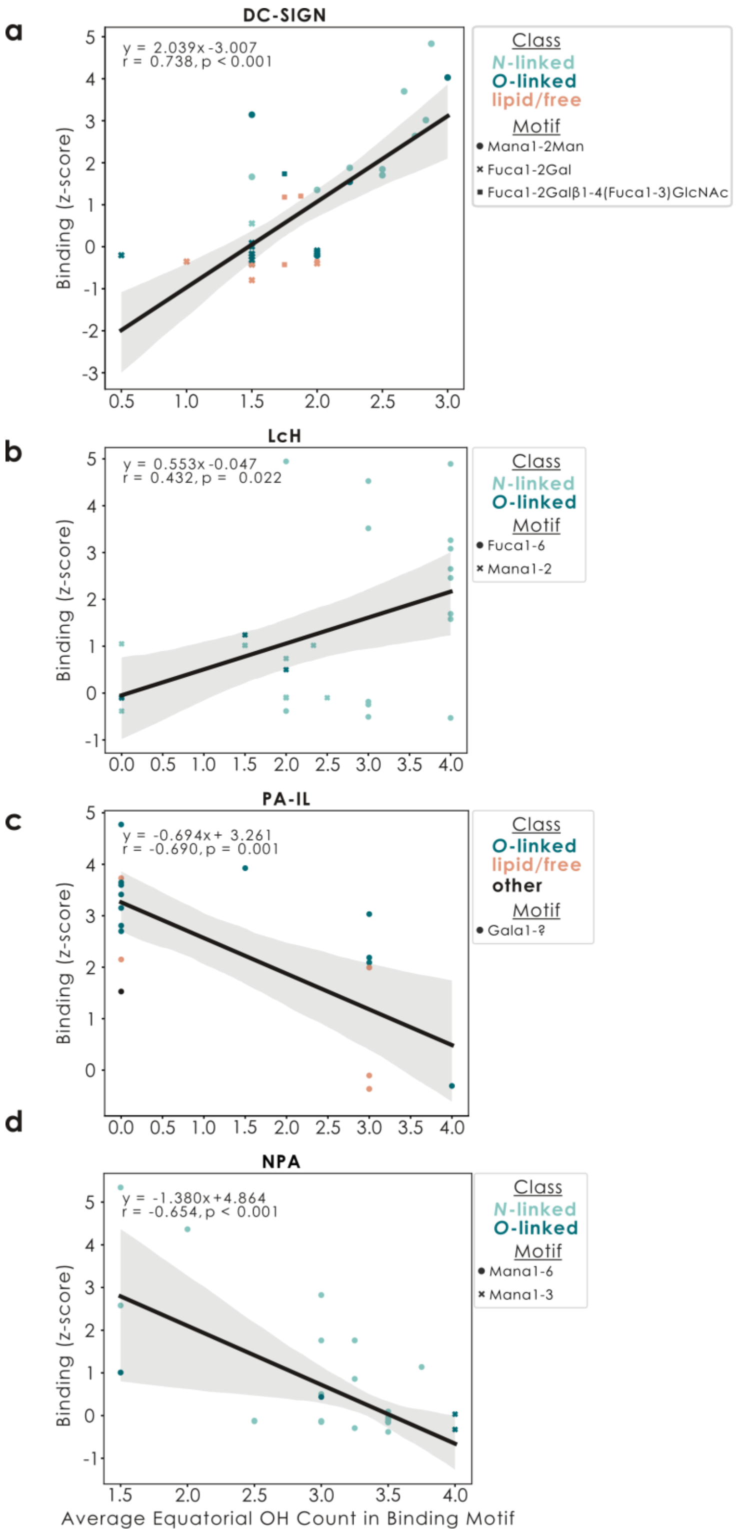

Supplementary Figure 11. Hydroxyl conformation of the binding motif affects lectin binding. a-d) For the lectins DC-SIGN (a), LcH (b), PA-IL (c), and NPA (d), we used the z-score transformed binding data from glycowork (v1.6) and correlated it with the average number of equatorial hydroxyl groups in the binding motif of the literature-known binding motifs in each glycan that (i) carried a binding motif, (ii) had binding data, and (iii) was deposited on GlycoShape. On top of the data points as a scatter plot (colored by glycan class), we then drew a linear least-squares regression line (line representing the mean and including 95% confidence band) as well as the regression equation, the Pearson's correlation coefficient r, and the p-value of a two-sided t-test of the regression coefficient against zero.

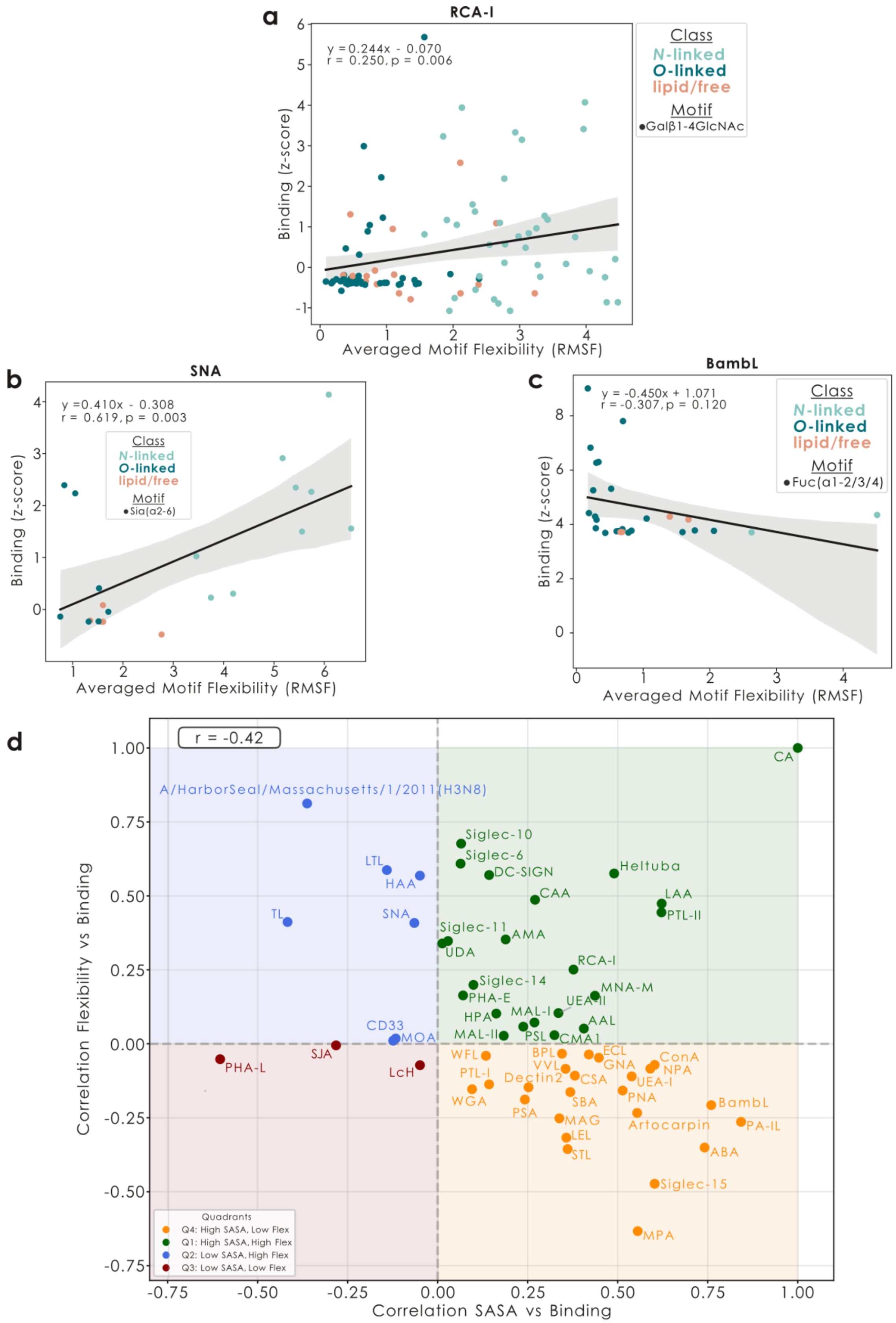

Supplementary Figure 12. Assessing the impact of glycan distance-flexibility on lectin binding. a-c) For the lectins RCA-I (a), SNA (b), and BambL (c), we present the correlation of lectin binding with distance-based glycan flexibility (analogous to torsion-based flexibility in Fig. 3). **d)** Overview of lectin binding correlation with distance-based flexibility, compared to torsion-based flexibility (in Fig. 4a).

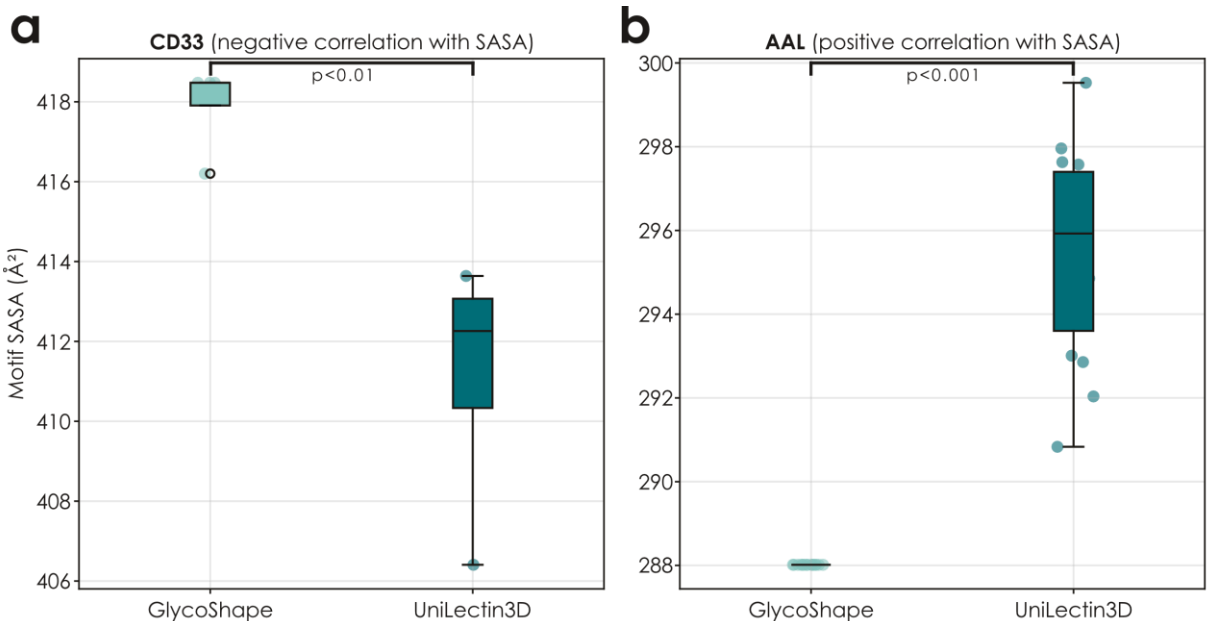

Supplementary Figure 13. Validating SASA preferences of lectins with UniLectin3D. a-b) For the lectins CD33 (a) and AAL (b), we analyzed the SASA values of all their co-crystal structures on UniLectin3D and compared this to the weighted average SASA value of the same binding motif in GlycoShape. Statistical testing included a two-tailed paired t-test (a: $p = 0.0078$; b: $p = 3.6e-12$), followed by Benjamini-Hochberg correction for multiple testing. Box plots: The line represents the median values, with box edges indicating quartiles, and whiskers indicating the remaining data distribution up to the 95% confidence interval. Individual data points are plotted on top of the box plots, with additional x-jitter for visibility.

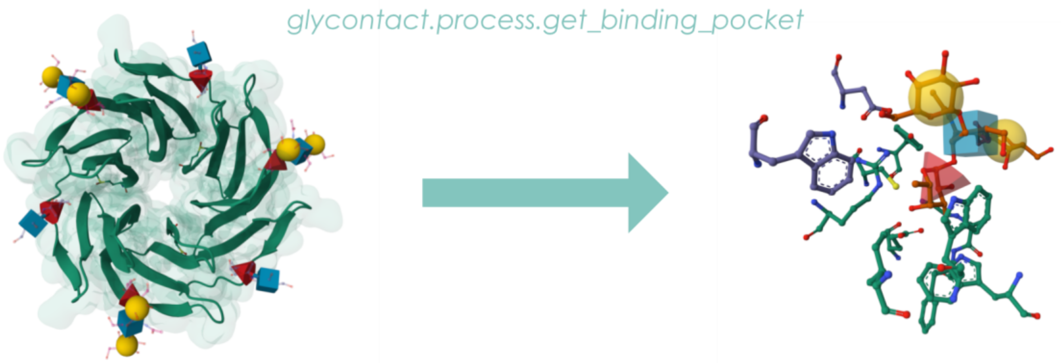

Supplementary Figure 14. Extracting binding pockets from lectin co-crystal structures. For the fucose-binding lectin BambL (PDB: 3ZW1), we show the full co-crystal structure that includes multiple glycans as well as the new PDB file that is generated by the *glycontact.process.get_binding_pocket* function. In the binding pocket, amino acids retrieved with the "binding_monosaccharide = Fuc" argument are colored green and amino acids making contacts (< 4 Å) with other monosaccharides are shown in blue.

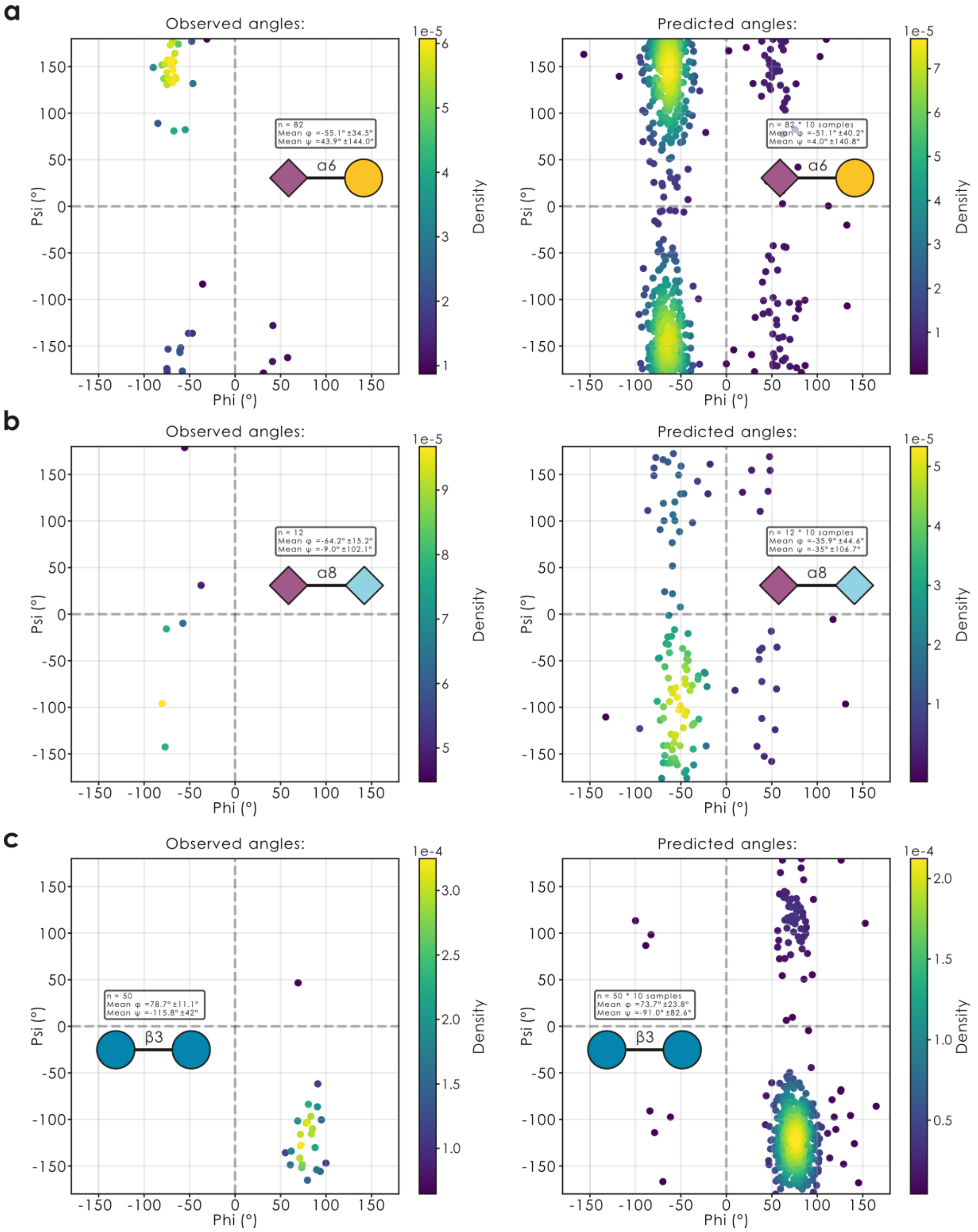

Supplementary Figure 15. Modeling torsion angles as multimodal von Mises distributions predicts seen and unseen torsion angles accurately. a-c) For the torsion angles of the disaccharides Neu5Acα2-6Gal (a; seen in training dataset), Neu5Acα2-8Neu5Gc (b; not seen in training dataset), and Glcβ1-3Glc (c; not seen in training dataset), we contrasted observed and predicted torsion angles with the *glycontact.visualize.ramachandran_plot* function. For predicted Ramachandran plots, we used a trained SweetNet-style model by sampling 10 points for each disaccharide sequence occurrence from the multimodal von Mises distribution created from predictions.

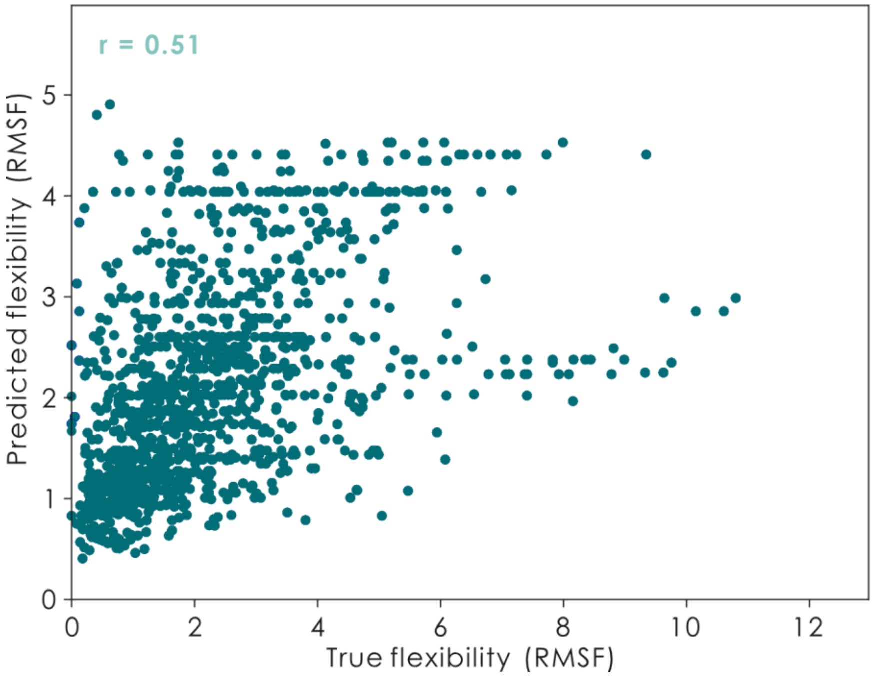

Supplementary Figure 16. Glycan flexibility predictions correlate with observed flexibilities. For all glycans in our test set, we extracted their monosaccharide-level flexibility as well as their predicted flexibility using the von Mises-SweetNet model. Shown is a scatter plot of predictions vs ground truths (in root mean square fluctuation or RMSF) as well as their correlation coefficient as Pearson's r.
