# Supplementary material for: GlyContact analyzes glycan 3D structures at scale
Source: Nat Commun. 2025 Dec 12;16:11136. doi: 10.1038/s41467-025-67590-y (PMC12706034; doi:10.1038/s41467-025-67590-y)
Supplement: Supplementary file 2 — Description of Additional Supplementary Files [file 41467_2025_67590_MOESM2_ESM.pdf]

Description of Additional Supplementary Files

File Name: Supplementary Data 1
Description: A list of the 717 glycans for which PDB structures from GlycoShape are included in GlyContact and in the analysis of this work.

File Name: Supplementary Data 2
Description: Lectin-glycan complexes extracted from UniLectin3D. For each complex, we note the PDB ID, the resolution of the structure in Ångström, the protein UniProt ID, and the glycan sequence present in the PDB coordinates in IUPAC-condensed nomenclature. We note that, for some glycans, several copies of the indicated sequence could be found in the PDB file (for instance in separate binding pockets of the lectin) and were extracted separately, leading to the total number of 1,437 lectin-glycan complexes.

File Name: Supplementary Data 3
Description: Used lectin binding specificities for our analyses. For each lectin, we provide its name, its binding specificities as a list of IUPAC-condensed motif sequences, and specifications for each monosaccharide in such motifs whether that monosaccharide had to be terminal ('t'), internal ('i'), or flexible ('f').

File Name: Supplementary Data 4
Description: Frequency of bound conformer by lectins from UniLectin3D. For lectin-glycan complexes in which we had the bound glycan represented in our GlycoShape structures, we retrieved the conformer frequency of the closest-matching conformer (via structural alignment) and display it here as a percentage.

File Name: Supplementary Data 5
Description: Co-crystal structures from UniLectin3D for lectins binding fucose-containing glycan motifs.

File Name: Supplementary Data 6
Description: Dataset used to train LectinOracle-struct. For glycans for which we had both glycan array binding data and 3D information, we obtained all their lectin binding data from glycowork (v1.6) and record it here, in the form of z-score transformed data.
